# Causal role of histone acetylations in enhancer function

**DOI:** 10.1080/21541264.2016.1253529

**Published:** 2016-10-28

**Authors:** Madapura M. Pradeepa

**Affiliations:** School of biological sciences, University of Essex, Colchester, UK

**Keywords:** chromatin, enhancers, gene expression, histone acetylation, H3 globular domain

## Abstract

Enhancers control development and cellular function by spatiotemporal regulation of gene expression. Co-occurrence of acetylation of histone H3 at lysine 27 (H3K27ac) and mono methylation of histone H3 at lysine 4 (H3K4me1) has been widely used for identification of active enhancers. However, increasing evidence suggests that using this combination of marks alone for enhancer identification gives an incomplete picture of the active enhancer repertoire. We have shown that the H3 globular domain acetylations, H3K64ac and H3K122ac, and an H4 tail acetylation, H4K16ac, are enriched at active enhancers together with H3K27ac, and also at a large number of enhancers without detectable H3K27ac. We propose that acetylations at these lysine residues of histones H3 and H4 might function by directly affecting chromatin structure, nucleosome–nucleosome interactions, nucleosome stability, and transcription factor accessibility.

## Introduction

**Figure 1.**
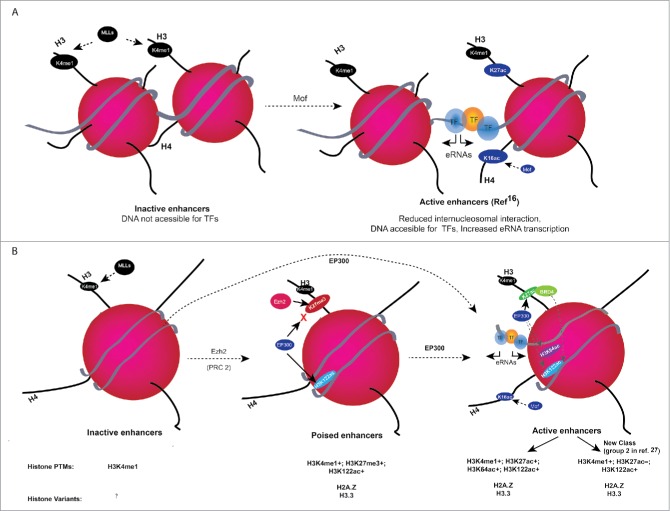
(A) H4K16ac role in increasing the accessibility of DNA to TFs basic amino acid residues at the histone H4 tail interaction with the acidic patches of H2A from neighboring nucleosomes, which is important for higher order chromatin folding. Mof-mediated acetylation of lysine 16 on the histone H4 (H4K16ac) tail disrupts the inter-nucleosomal interaction *in vitro*, which disrupts the higher order chromatin structure and might also increase the accessibility of TFs to DNA and increased eRNA transcription. (B) Working model for histone H3 globular acetylations at regulatory elements. Monomethylation of H3K4 residues (H3K4me1) away from transcription start sites (TSSs) is widely used to identify enhancers. Inactive enhancers are H3K4me1 positive, and they lack detectable level of histone acetylations. Poised enhancers are enriched for H3K27me3 along with H3K4me1 and H3K122ac. At active enhancers, EP300 acetylates nucleosomes at H3K27, H3K64, H3K122, and Mof acetylates H4K16. H3K122ac co-localizes with H2A.Z-containing nucleosomes, suggesting that H3K122ac might contribute to destabilization of nucleosomes at active and poised enhancers. Working model shows that EP300-mediated H3 globular domain acetylations lead to increased accessibility of nucleosomal DNA for TFs by reducing the affinity between histone and DNA. H3 globular acetylation facilitates the remodeling of nucleosomal DNA and contributes to destability of labile nucleosomes and eRNA transcription. Presence (+) or absence (−) of histone modifications, histone variants at enhancer classes is given below.

Histone post-translational modification (PTM) status, DNA methylation, transcription factor (TF) binding, and DNA accessibility profiles are extensively used to understand the gene regulatory mechanisms in various cell and tissue types. Although much progress has been made in genome-wide profiling of chromatin modifications, occupancy of TFs, and coactivators, most of the data is still correlative, and it is difficult to delineate the importance of each of these dynamic features in regulating chromatin structure and/or gene expression (reviewed in ref.^1^). Most histone tail PTMs function by recruiting chromatin proteins that have distinct domains that can ‘read’ specific histone modifications, which then affect various chromatin-mediated processes like transcription, DNA repair, and RNA processing.^2–6^ An increasing number of such proteins have been identified using chromatin proteomic techniques (highlighted in ref.^7^). Unlike histone H3 tail PTMs that depend on these effector proteins for their function, H4 tail modifications—H4K16ac and H4K20me—are known to directly affect chromatin structure by altering inter-nucleosomal interactions *in vitro* (Fig. 1A).^8-10^ PTMs can also occur at the histone globular domain that is also called histone fold domain. PTMs at the globular domain, especially at the lateral (outer) surface of the histone octamer, can directly affect nucleosomal stability and chromatin structure by altering the histone–DNA interactions (Fig. 1B).^11-13^

## Use of histone modifications to differentiate enhancers from promoters

A large number of efforts have been made to map various histone modifications using chromatin immunoprecipitation followed by deep sequencing (ChIP-seq) in a variety of cell types and organisms. Analysis of this data shows that the enrichment of most histone tail modifications correlate or anti-correlate with gene expression levels, and also that they are enriched at particular genomic features. For example, it is clear that H3K4me3 is present at promoters, whereas H3K4me1 is enriched at enhancers. This high H3K4me1 to H3K4me3 ratio is widely used to distinguish enhancers from promoters (reviewed in Ref.^14^). Along with H3K4 methylation, active promoters and enhancers are enriched with histone acetylations, such as H3K27ac and H4K16ac.^15-19^ Combinations of H3K4me1, H3K27ac, and H3K27me3 are widely used to classify active enhancers (H3K4me1 and H3K27ac positive) from inactive (H3K4me1 positive and H3K27ac negative) or poised enhancers (H3K4me1 and H3K27me3 positive).^15,17,20^

## Role of histone acetylations in regulation of gene expression

The functions of most histone tail modifications depend on effector proteins that recognize these modifications and bind to them. Exception to that is acetylation at H4K16 and methylation at H4K20, which have direct impacts on inter-nucleosomal interactions.^8,10^ The H4 tail has been shown to interact with the acidic patch of H2A in the adjacent nucleosomes, and H4K16ac inhibits 30-nm chromatin fiber formation by destabilizing the inter-nucleosomal interactions (Fig. 1A).

Similarly, PTMs located on the lateral surface of the histone octamer can directly alter contacts between histones and nucleosomal DNA and affect chromatin structure directly (reviewed in ref^21^). Acetylation of histone H3 at lysine 56 (H3K56ac) by EP300 is associated with DNA unwrapping from the nucleosome and has been implicated in chromatin assembly and genome stability.^22,23^ Although this modification is abundant and better studied in yeast, H3K56ac is present at very low levels in mammals and shown to be enriched at regulatory regions in human stem cells.^24^ Recently, two more lysine residues at the histone H3 globular domain, K122 and K64, have been shown to be acetylated by EP300.^11,12^

Both H3K64ac and H3K122ac are located at the lateral surface of the histone octamer. H3K64ac, a modification at the lysine residue at the start of the first α-helix in the histone fold domain, destabilizes nucleosomes and facilitates nucleosome dynamics *in vitro*.^11^ The H3K122 residue is located at the dyad axis where only one DNA fiber binds, and histone binding to DNA is at its highest strength (reviewed in ref.^25^). Rob Schneider's group has elegantly shown that H3K122ac directly impacts histone–DNA binding in the dyad axis. Unlike acetylation on histone tails, H3K122ac is sufficient to stimulate transcription *in vitro* from chromatinized templates^12^ and promote nucleosome disassembly^26^ (Fig. 1B). We have shown the presence of H3K122ac at promoters of expressed genes, and also at poised genes along with H3K27me3, H2A.Z, and H2A.Zac, which are known to be associated with poised genes.^27-29^ Our H3K122ac ChIP-seq data together with data from *in vitro* transcription assays and induction studies done in *Schizosaccharomyces pombe* suggest the causal role of H3K122ac in rapid activation of poised genes upon differentiation signaling.^12,27^

Although H3K64ac and H3K122ac are shown to be acetylated by EP300 acetyl transferase.^11,12^ Recently, bromodomain-containing protein 4 (BRD4), a chromatin reader protein that recognizes and binds acetylated histones, also has been recently shown to possess acetyl transferase activity. BRD4 also suggested to acetylate several lysine residues of histone H3 and H4, including H3K122.^30^ It has been proposed that BRD4 increases transcription by facilitating the eviction of nucleosomes by catalyzing the acetylation at H3K122. It remains to be investigated whether BRD4 can directly bind acetylated H3K122 or is recruited through histone tail acetylations. However, absence of histone tail acetylations at many H3K122ac sites suggests that there might be a histone acetylation independent mechanism of recruitment for BRD4 at these sites.

## Complex pattern of histone modification at enhancers

Although H3K4me1, H3K27ac, and EP300-binding profile are widely used to predict enhancer activity,^15,17,20^ it is becoming increasingly clear that this combination of histone marks and EP300 co-activator binding may not identify all active enhancers.^16,27,31,32^ We have previously shown that acetylation of Lysine 16 of histone H4 (H4K16ac) marks active enhancers that are associated with pluripotency-associated genes in mouse embryonic stem cells. Interestingly, there are also some active enhancers, which are H3K27ac negative but are marked with H4K16ac.^16^ KAT8/MOF which acetylates H4K16,^16^ and the ATAC complex containing Gcn5/PCAF, and H3K9ac, which is preferentially catalyzed by Gcn5/PCAF have also been detected at enhancers, suggesting a wider role of different lysine acetyl transferase complexes in enhancer function.^16,31,33^

Co-occurrence of H3 globular acetylations, H3K122ac and H3K64ac, with H3K4me1 at enhancer elements suggested their role in enhancer function. Moreover, we suggest H3K122ac can be used to identify active enhancer elements more robustly than the widely used H3K27ac mark, which misses a whole new class of enhancers.^27^ Interestingly, H3K64ac is enriched at highly active enhancers and also at long stretches of highly active enhancers so-called super-enhancers (SEs).^27,34^ SEs are known to transcribe higher levels of eRNAs compared to typical enhancers.^35^ It is reasonable to assume that H3K64ac might contribute to transcription of eRNAs at highly active enhancer elements, including SEs. High level of H3K64ac but not H3K122ac at these SEs suggests that although these two globular domain modifications are present at regulatory elements, they might have different functional mechanisms *in vivo*. It will be interesting to study whether simultaneous acetylations at both H3K122 and H3K64 further increase eRNA transcription and contributes to enhancer activity.

## Unique features of H3K122ac positive enhancers

Although high levels of H3K64ac and H3K122ac are detected at active enhancers as defined by the presence of H3K27ac, H3K122ac is also found at H3K27ac negative enhancers, a subset of which are H3K27me3 positive. H3K27me3 and H2A.Z are known to colocalize across polycomb-repressed genes and enhancers.^15,28,29^ Co-occurrence of H3K122ac with H3K27me3 and H2A.Z at both poised promoters and at H3K27ac negative enhancers suggests that subset of H3K122ac positive enhancers are poised. The presence of H3K122ac, H2A.Z at poised promoters and enhancers hints at a causal role of H3K122ac and H2A.Z containing labile nucleosomes in increasing the accessibility of TFs for rapid induction of transcription upon differentiation signaling.^12,27^

## DNA methylations at enhancers

DNA methylation is associated with gene repression, and CpG islands located at promoters are generally unmethylated, in contrast to those at gene bodies of expressed genes that are methylated.^36^ Intriguingly, recent studies also show a higher level of DNA methylation at active enhancers that transcribe eRNAs, although the significance of this is not known.^37,38^ Like gene body methylation, enhancer methylation has been shown to be dependent on transcription and H3K36me3-mediated recruitment of DNMT3A/DNMT3B.^39^ This new observation of eRNA-transcription-dependent DNA methylation at enhancers suggests that enhancers and genes share similar chromatin features. Increasing pieces of evidence show that like promoters, chromatin features at enhancers are more complex than previously thought (Fig. 1), and it is important to consider the combinatorial features of enhancers to decipher the enhancer activity in a given cell type.^16,27^

Recent studies show correlation of active enhancers (H3K27ac positive) with DNA methylation at enhancers.^37^ DNA methylation is known to inhibit the CTCF binding at many sites (reviewed in^40^). We speculate that subclass of H3K122ac enhancers has a higher level of CTCF and other architectural proteins than H3K27ac enhancers; these multifunctional proteins might contribute to a unique mechanism of enhancer action. Further investigations are needed to improve our understanding of interplay among TFs, DNA methylation, CTCF and its interacting partners at H3K122ac enhancers.

## Enhancer RNAs (eRNA), their process of transcription in enhancer mechanism

Enhancer activity is correlated with bidirectional transcription from enhancer elements by RNA Polymerase II, producing a class of non-coding RNAs called eRNAs^41-43^ which are degraded by exosome complexes.^44^ There is a clear association of enhancer elements with bidirectional transcription as measured by capped analysis of gene expression (CAGE) tags,^43^ and it has been suggested that such bi-directional unstable transcripts might be the best way to catalog active enhancers in a particular cell-type. However, recent computational analysis argues against the specificity of bidirectional transcription in identifying active enhancer elements, as it appears to arise due to promiscuous RNA Pol II activity at accessible chromatin in general and not just that specific to enhancers.^45^ Importance of eRNAs in enhancer function is still controversial; however, there are few eRNAs that are shown to be functional in positively regulating target genes.^46,47^ Some long noncoding RNAs (lncRNAs) such as Hottip are also shown to have enhancer-like function.^48,49^

## Causal role of histone H3 globular acetylations at enhancers

The functional significance of histone marks at enhancers is not known (reviewed in ref.^1^). Most histone tail modifications depend on effector proteins, which bind to modified histone tails. Bromodomain-containing proteins—e.g. BRD2, 3, and 4—bind to acetylated histone tail residues at promoters as well as enhancers and have been shown to promote transcription of protein coding genes and eRNAs.^50^ Acetylation at H3 globular domain residues, H3K64 and H3K122, located at the lateral (outer) surface of the histone octamer can directly destabilize nucleosomes and stimulate transcription.^21^ Enrichment of these modifications at active promoters and enhancers suggests their causal role in stimulating transcription of genes and eRNAs. Our recent genome-wide studies in ESCs and cancer cells support the data from previous *in vitro* experiments and induction experiments done in *S. pombe*.^11,12^ Together, we suggest a causal role of these H3 globular domain acetylations in stimulating transcription at both promoters and enhancers.^11,12,27,51^ Intriguingly, we detect low level of CAGE tags in H3K122ac positive enhancers, which lack detectable level of histone tail acetylations. However, these enhancers indeed transcribe bidirectional eRNA, but are degraded by exosome complexes.^27,52^ This data shows that many of the bidirectional eRNAs at new class of H3K122ac enhancers are either low abundant or rapidly degraded by exosome complexes; it is possible to miss identification of some of the H3K122ac positive active enhancers using CAGE based assays.

## Importance of labile nucleosomes at enhancers

H3K122ac is detected at nucleosomes with histone variants H3.3 and H2A.Z at promoters and enhancers,^12,27^ suggesting that the K122 residue might be acetylated on H3.3 molecules. Nucleosomes with these double histone variants and H3K122ac are known to be particularly unstable,^26,53^ and such labile H3.3/H2A.Z nucleosomes are shown to occupy regulatory elements which are widely regarded as ‘nucleosome free.’^53,54^ We speculate that acetylation of H3K122 contributes to the destabilization of H3.3/H2A.Z nucleosomes at promoter and enhancer elements and that this will aid the binding of TFs.

## Future perspectives

Recent functional genomics data suggests that there are many more gene regulatory elements than previously thought (ENCODE consortium). Although a large number of disease-associated single nucleotide polymorphisms (SNPs) map to enhancer elements, a lack of information about gene-specific function of enhancers means that the causal role of most SNPs in disease is not known. Increasing evidence suggests that the accessible chromatin structure caused by labile nucleosomes and/or the act of transcription at enhancers might have a causal role in enhancer activity. Recent development in genome editing tools like TALE and CRISPR Cas9 and their versatility will no doubt be useful in deciphering the importance of DNA methylation, histone modifications, or the act of eRNA transcription at enhancers.^27,55-58^

The role of histone modifications in enhancer function is generally not clear, and it has been challenging to find out the enhancer specific functions for histone modifications. Although H3K122ac is enriched at active enhancers along with H3K27ac, a subset of H3K122ac-marked novel enhancers is also enriched for H3K27me3, a marker of poised enhancers, suggesting a role for H3K122ac in maintaining accessible chromatin at both active and poised regulatory elements.^27^ Investigations into the specific recruitment mechanisms of protein complexes that modify histone and non-histone proteins, together with the understanding of the importance of reader proteins that binds to these modifications will be useful in deciphering the importance of these PTMs in enhancer function.

The presence of H3 globular domain acetylations at the lateral surface of the histone octamer together with histone tail acetylations suggests that there are both effector dependent and independent functions for these modifications in enhancer function. Further investigations are needed to appreciate the functional importance of these complex and highly dynamic chromatin features at enhancers in maintaining the chromatin structure and regulation of gene expression.

In summary, it is important to address the cause/consequence relationship of dynamic features at enhancers like open chromatin, histone PTMs, histone variants, the process of bidirectional transcription, and DNA methylation in order to understand enhancer mechanisms. Future work will potentially unveil the complex interplay between the process of eRNA transcription, histone modifications, and protein complexes present at enhancer elements and their contribution to the distal regulation of gene expression.
